# Therapeutic opportunities for targeting microRNAs in cancer

**DOI:** 10.1186/2052-8426-2-30

**Published:** 2014-10-03

**Authors:** Molly A Taylor, William P Schiemann

**Affiliations:** Oncology iMed, AstraZeneca R & D, Room 33F83/7 Mereside, Alderley Park, Macclesfield, SK10 4TG UK; Case Comprehensive Cancer Center, Case Western Reserve University, Wolstein Research Building, Room 2131, 2103 Cornell Road, Cleveland, OH 44106 USA

**Keywords:** Antisense oligonucleotides, Biomarkers, Chemotherapeutics, Locked nucleic acids, MetastamiR, Metastasis, microRNA, OncomiR

## Abstract

MicroRNAs (miRNAs) are small noncoding RNAs that can function as either powerful tumor promoters or suppressors in numerous types of cancer. The ability of miRs to target multiple genes and biological signaling pathways has created intense interest in their potential clinical utility as predictive and diagnostic biomarkers, and as innovative therapeutic agents. Recently, accumulating preclinical studies have illustrated the feasibility of slowing tumor progression by either overexpressing tumor suppressive miRNAs, or by neutralizing the activities of oncogenic miRNAs in cell- and animal-based models of cancer. Here we highlight prominent miRNAs that may represent potential therapeutic targets in human malignancies, as well as review current technologies available for inactivating or restoring miRNA activity in clinical settings.

## Review

### Introduction

The war against cancer commenced in 1971 with the enactment of the National Cancer Act and continues unabated today against this most common killer of American men and women between the ages of 40-79 [1]. Indeed, the American Cancer Society estimates that 25% of all deaths in the United States can be directly attributed to cancer, which is anticipated to account for nearly 600,000 deaths and 1.7 million new invasive cancer cases in 2014. Despite this rather bleak outlook, the collective efforts of science and medicine have nevertheless dramatically reduced the annualized cancer death rate by 20% over the last two decades, thereby sparing the lives of more than 1.3 million Americans [1]. Building upon this success will require the development of new diagnostic and prognostic tests, as well as the creation of novel targeted chemotherapies derived from enhanced knowledge of the molecular mechanisms coupled to tumorigenesis and metastatic progression.

The central dogma of molecular biology states that the transfer of genetic information within cells transpires sequentially from DNA to RNA to proteins, whose coding sequences comprise a paltry 1.5-2% of the human genome [2, 3]. Although genetic and epigenetic aberrations that occur in components of the central dogma clearly elicit disease development in humans, recent findings also point to a prominent role for non-protein-coding regions of the genome in regulating cell and tissue homeostasis, as well as in contributing to the formation of human tumors. Included amongst the various classes of noncoding RNAs are members of the PIWI-interacting RNA (piRNA) family, the small nucleolar RNA (snoRNA) family, the large intragenic noncoding RNA (lincRNA) family, the long noncoding RNA (lncRNA) family, and the transcribed ultraconserved regions (T-UCR) family of the lncRNAs [3–5]. However, the best characterized and most extensively studied class of noncoding RNAs belong to the family of microRNAs (miRNAs), which play essential functions during embryogenesis and tissue development, and during cell differentiation, proliferation, and survival [4, 5]. As a group, miRNAs are small (17-27 nucleotides) noncoding RNAs that govern gene expression in a post-transcriptional manner by binding directly to the 3′UTRs of target mRNAs, thereby repressing their translation or inducing their degradation [6]. In doing so, miRNAs have been ascribed as being potent tumor suppressors in normal cells, and as being robust tumor promoters in developing and progressing carcinomas [7, 8]. Moreover, miRNAs are frequently located at fragile genome sites or regions that are frequently amplified or deleted in human cancers [9]. Indeed, emerging evidence indicates that miRNAs function as a molecular rheostats that serve in fine-tuning cell signaling pathways [10, 11], doing so by modulating the expression of large numbers of genes and, consequently, impacting the flux through essential regulatory nodes of vast signaling networks. These functional characteristics underlie the belief that targeting and manipulating either the expression or activity of miRNAs may provide novel inroads to treat human cancers, a supposition currently being evaluated in phase I clinical trials for MRX34 (a miR-34 mimetic) against late-stage hepatocellular carcinomas and a variety of lymphomas ([5]; ClinicalTrials.gov number, NCT01829971), and in phase II clinical trials for miR-122 antagonists against hepatitis ([12]; ClinicalTrials.gov number, NCT01200420). In the succeeding sections, we review recent findings pertaining to how miRNA expression transpires in normal cells, and to how these events become dysregulated in malignant cells, leading to their acquisition of metastatic and chemoresistant phenotypes. Finally, we discuss current therapeutic strategies aimed at targeting inappropriate miRNA expression and activity in developing and progressing human carcinomas.

### miRNA biogenesis

**Figure 1:**
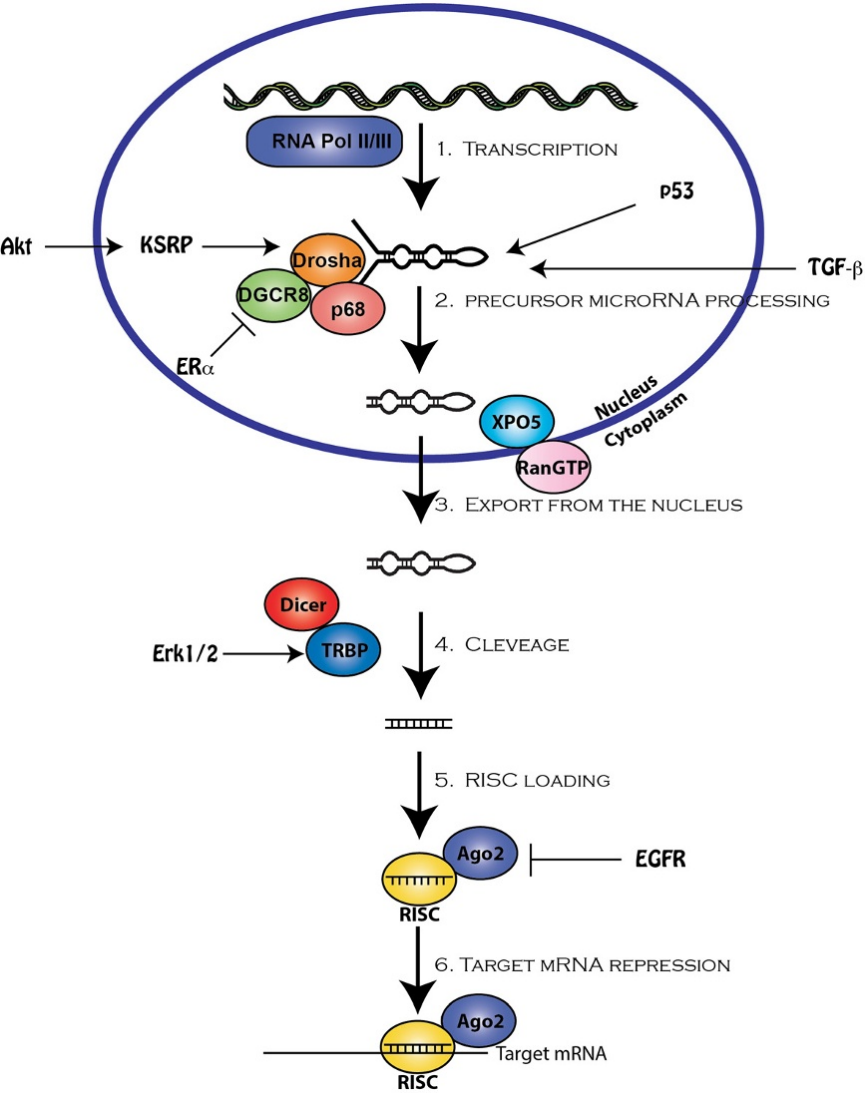
**miRNA biogenesis.** miRNAs are transcribed by RNA polymerase II or III (Pol II/III) to produce primary transcripts (pri-miRNAs), which are subsequently processed and cropped *via* the actions of the Drosha-DGCR8 complex, which together with the RNA helicases p68 and p72 generate the formation of precursor miRNAs (pre-miRNAs). Activation of the TGF-β, Akt/PI3K, and p53 signaling systems have all been shown to promote the processing of specific pri-miRNAs, while stimulation of the ER-α signaling system is capable of repressing pri-miRNA processing. Once processed, pre-miRNAs hairpins are exported from the nucleus by exportin-5 (XPO5)-RanGTPase complexes, and are subsequently cleaved by Dicer:TRBP complexes, thereby producing mature oligonucleotide duplexes. The rate at which Dicer cleaves pre-miRNAs is greatly enhanced by the phosphorylation of TRBP by Erk1/2. At the completion of pre-miRNA cleavage, Dicer serves with Argonaute (Ago) 2 in loading mature miRNAs into RNA-induced silencing complexes (RISCs), thereby silencing target mRNA expression through mRNA cleavage, translational repression, or deadenylation. Hypoxic conditions resulting in EGFR activation have been shown to induce the phosphorylation of Ago2, leading to diminished maturation reactions of select miRNAs.

miRNAs are transcribed in the nucleus by the actions of RNA polymerases II or III to produce a long primary miRNA transcript (pri-miRNA), which subsequently associates with the Drosha-microprocessor complex that houses the *(i)* RNase III enzyme, Drosha, *(ii)* DGCR8 (DiGeorge critical region 8), and *(iii)* RNA helicases p68 and p72 (*see* [13]). In addition, the KH-type splicing regulatory protein (KSRP) can also associate with the microprocessor complex to regulate the biogenesis of a subset of miRNAs operant in regulating cell differentiation, proliferation, and survival [14]. Endonucleolytic cleavage of pri-miRNAs by the microprocessor complex leads to formation of precursor miRNAs (pre-miRNA), which exist as hairpin loop structures of ~60-70 nucleotides in length that are exported from the nucleus by the actions of Exportin-5, with assistance from Ran-GTPases [15]. Upon gaining access to the cytoplasm, the pre-miRNA is further processed and cleaved by the Dicer/TRBP (Tar RNA-binding protein) complex to produce a mature ~21 nucleotide RNA duplex [16], which together with Argonaute (Ago2) proteins is loaded into the RNA-induced silencing complex (RISC). Interestingly, the orientation of mature miRNAs as they are loaded into RISC complexes is governed by their 5′-antisense ends, which possess enhanced flexibility and lower internal stabilities that facilitate miRNA unwinding and strand retention by active RISCs [17]. Finally, the extent of sequence complementarity between the 5′-seed region of the loaded miRNA with that of its target mRNA 3′UTR largely dictates whether individual mRNAs are inactivated *via* cleavage, translational repression, or deadenylation (Figure 1; [13]). As will be discussed below, recent studies clearly demonstrate the importance of oncogenic signaling systems to impinge upon multiple steps of the miRNA biogenesis pathway, resulting in the aberrant expression and activity of miRNAs during tumorigenesis [18, 19].

### Aberrant miRNA biogenesis in carcinomas

Although major progress has been achieved in terms of understanding the fundamental mechanisms whereby miRNAs are synthesized and processed, considerably less knowledge exists regarding the specific intracellular pathways and effector molecules coupled to the regulation of miRNA biogenesis. Recently, administration of either transforming growth factor-β (TGF-β) or bone morphogenetic protein-4 (BMP-4) to pulmonary artery smooth muscle cells (PASMCs) was observed to dramatically elevate their levels of mature miR-21 independent of any alterations in the transcription of pre-miR-21 [20], suggesting that these multifunctional cytokines drive the processing of miR-21, not its transcription. Accordingly, receptor-regulated Smad transcription factors for TGF-β (Smads 2 and 3) and BMP-4 (Smads 1, 5, and 8) were found to associate with Drosha complexes by interacting with the RNA helicase, p68 [20], a reaction dependent upon the presence the Smad-binding elements (SBEs) located in the stem regions of ~20 pre-miRNAs known to be responsive to TGF-β/BMP-4 stimulation [21]. Indeed, engineering SBE sequences into stem loop structures was sufficient to confer TGF-β/BMP-4-mediated processing of pre-miRNAs to yield their mature products (Figure 1; [21]). It should be noted that the ability of transcription factors to drive miRNA processing is not unique to TGF-β/BMP-regulated Smads, but is instead a widespread phenomenon as evidenced by the fact that ~45% of all pre-miRNAs harbor one or more consensus binding sites for transcription factors [22]. Indeed, similar to Smads, the tumor suppressor p53 has also been shown to interact with the Drosha complex through p68, resulting in enhanced Drosha processing of pre-miRNAs coupled to DNA damage responses and anticancer activities (*e.g.,* miR-16-1, miR-143, miR-145; [23]). Additionally, hyperactivation of the Ras/ERK1/2, PI3K/Akt, and ATM/DNA damage pathways are commonplace in carcinoma cells and have recently been shown to promote the phosphorylation of TRBP, which enhances Dicer cleavage activity, and of KSRP, which enhances pri-miRNA processing reactions [24–27]. Thus, one mechanism whereby oncogenic signaling systems promote tumor development and metastatic progression transpires through upregulated miRNA processing and its associated inactivation of tumor suppressing genes and pathways.

In stark contrast to aforementioned mechanisms that underlie the increased processing of miRNAs, recent findings also observed significant reductions in miRNA processing in response to oncogenic signals. For instance, estradiol-mediated activation of estrogen receptor-α (ER-α) drives the association of this steroid receptor with p68, resulting in widespread inhibition of pri-miRNAs by Drosha complexes [28]. Likewise, hypoxic stress enables the epidermal growth factor receptor (EGFR) to phosphorylate Ago2 and prevent its binding to Dicer, thereby inhibiting the processing and maturation of tumor suppressive pre-miRNAs (*e.g.,* miR-31, miR-192, and mir-193-5p; [29]). Future studies need to further elucidate the molecular mechanisms whereby oncogenic signaling pathways converge on the miRNA biogenesis network, particularly with respect to defining the sequence and specificity of these aberrant interactions, which may aid in discovering novel miRNA-based targeted therapies to alleviate carcinoma development and metastatic progression.

### Regulation of miRNAs in carcinomas

The “Hallmarks of Cancer” were eloquently proposed by Hanahan and Weinberg to describe the essential features ingrained in the physiology of all malignant cells that drives their growth and dissemination. Indeed, included amongst the traits displayed by all carcinoma cells is their ability to *(i)* activate proliferative and replicative immortality programs, while simultaneously inactivating antigrowth programs; *(ii)* resist apoptotic programs; and *(iii)* stimulate angiogenic, invasive, and metastatic programs [30, 31]. Importantly, miRNAs play active roles in modulating all of these physiological processes in carcinomas, typically acting on multiple pathways and programs to elicit disease development. Identifying and understanding how miRNAs regulate the balance of and flux through these signaling systems may provide novel opportunities to design and implement miRNA-directed therapies to treat human malignancies. In the succeeding sections, we highlight a variety well-characterized and essential miRNAs operant in driving tumorigenesis based on their classification as oncomiRs (*i.e.,* tumor promoting miRNAs), tumor suppressive miRNAs, or metastamiRs (*i.e.,* metastasis promoting miRNAs) (Table 1). It should be noted that this discussion is by no means comprehensive, and as such, readers desiring more extensive summaries are directed to several recent reviews [5, 32, 33].

**Table 1:** **Identity and function of cancer-associated miRNAs**

microRNA	Disease setting	Targets	Hallmark (s) of cancer
oncomiRs			
miR-17 ~ 92 Family	Lymphoma and some solid tumors [36]	PTEN [37, 38], Bim [7, 37], p21 [40, 41], TSP1 [42], CTGF [42]	Resisting cell death
			Sustaining proliferative signaling, and inducing angiogenesis
miR-21	Glioblastoma [44], breast, colon, lung, pancreas, prostate, chronic lymphocytic leukaemia, Diffuse Large B-Cell Lymphoma, acute myeloid leukaemia and others [45, 46]	PTEN, p63, PDCD4, and RECK [46]	Sustaining proliferative signaling, resisting cell death, activating invasion and metastasis
miR-155	Lymphoma [50, 51], breast, colon, lung, pancreatic, and thyroid cancer [52]	RhoA [53], SOCS1 [54], FOXO3a [55]	Sustaining proliferative signaling, resisting cell death, activating invasion and metastasis
**Tumor suppressor miRs**			
miR-15a ~ 16-1	CLL [56], multiple myeloma, mantle cell lymphoma, and prostate cancers [57]	Bcl2, cyclin D1, and WNT3A [59]	Evading growth suppressors, Resisting cell death
let-7	Lung [61], colon, breast, and ovarian cancers [62]	Ras [66], HMGA2 [67], c-Myc [65]	Evading growth suppressors
miR-29 Family	Lung cancer, melanoma and myeloid leukemia [75]	CDK6 [70, 71], Ppmid [68], osteonectin [72], Mcl1 [73], Bcl2 [74], DNMT3a [75], and extracellular matrix genes [76].	Sustaining proliferative signaling, Enabling replicative immortality, Resisting cell death
miR-34	Breast, lung, colon, kidney, bladder, pancreatic cancer, and melanoma [84]	Bcl2, cyclin D1, Cyclin E2, CDK4, CDK6, c-Myc , MET, N-Myc, and SIRT1 [78–83]	Resisting cell death, Evading tumor suppressors, Enabling replicative immortality.
**metastamiRs**			
miR-200 Family	Breast cancer [91, 92]	Zeb1, Zeb2 [93], and Sec23a [95]	Activating invasion metastasis
miR-9	Breast [96] and colon [98] cancer	E-cadherin [96], LIFR, Cyclin D1, and Ets1 [97, 98]	Activating invasion metastasis
miR-31	Breast cancer [99]	ITGA5, RDX, RhoA and WAVE3 [100, 101]	Activating invasion metastasis
miR-10b	Breast [102, 103] and esophageal cancer [104]	HOXD10 [102]	Activating invasion metastasis
miR-181a	Breast cancer [108]	Bim [108], ATM [109]	Activating invasion metastasis

### oncomiRs

#### miR-17-92 cluster

The best characterized oncomiR is the polycistronic miR-17-92 (also known as oncomiR-1), whose expression from chromosome 13 is driven by c-Myc and results in the production of six mature miRNAs, namely miR-17-5p, miR-18a, miR-19a, miR-20a, miR-19b-1, and miR-92a-1 [34]. Additionally, two miR-17-92 paralogs have been identified: *(i)* the miR-106a-363 cluster, which is located on the X chromosome and houses miR-106a, miR-18b, miR-20b, miR-19b-2, miR-92a-2, and miR-363, and *(i)* the miR106b-25 cluster, which is located on chromosome 7 and houses miR-106b, miR-93, and miR-25 [35]. Consistent with its designation as an oncomiR, the location of the miR-17-92 cluster on chromosome 13 is found within a genomic region that is frequently amplified in a variety of human tumors, including diffuse B-cell, follicular, and Burkit’s lymphomas, and lung carcinomas [36]. Initial studies demonstrated that overexpression of mir-17-92 in an Eμ-Myc transgenic mouse model of Burkit’s lymphoma was sufficient to accelerate the tumorigenic process by suppressing apoptosis [34]. Indeed, the anti-apoptotic effects of this oncomiR cluster in haematopoietic malignancies can be ascribed to its targeting of the Bim and PTEN tumor suppressors [37–39]. With respect to solid tumors, the miR-17-92 cluster has been shown to induce proliferation, as well as augment angiogenesis through its ability to target p21 [40, 41], thrombospondin-1 (TSP1), and connective tissue growth factor (CTGF; [42]). Along these lines, the miR-106b-25 cluster was observed to drive mammary tumorigenesis and epithelial-mesenchymal transition (EMT) programs by targeting the inhibitory Smad, Smad7, thereby enhancing oncogenic TGF-β signalling [43]. Although the exact physiological function and degree of functional redundancy possessed by individual miRNAs of the mir-17-92 cluster remains to be fully elucidated, recent evidence indicates that these miRNAs do in fact exhibit a combination of unique and overlapping functions that coalesce in regulating embryogenesis and tissue development, as well as in driving tumorigenesis [39]. Future studies clearly need to expand our understanding of which mRNAs are targeted by the miR-17-92 cluster and its paralogs, thereby providing potential inroads for the development of novel therapeutics capable of alleviating the oncogenic activities of these potent oncomiRs.

#### miR-21

Although miR-21 was originally identified as an oncomiR in glioblastomas [44], subsequent large-scale miRNA expression profiling analyses of 540 tumors spanning 6 distinct types of cancer (*e.g.,* lung, breast, stomach, prostate, colon, and pancreas) demonstrated that miR-21 was upregulated in all tumor cohorts [45]. More recently, upregulated miR-21 expression has also been detected in lymphoid malignancies, including chronic lymphocytic leukemia, diffuse large B cell lymphoma, and acute myeloid leukemia (*see* [46]). Mechanistically, hyperactivation of the Ras/MAP kinase and NF-κB signaling systems in carcinomas elicits transcription of pri-miR-21 [47], whose processing to maturity is dramatically enhanced by the TGF-β signaling system [20]. Additionally, robust miR-21 expression has been observed to target a variety of essential tumor suppressors, including PTEN, p63, PDCD4, and RECK, which serve in promoting the proliferation, survival, and metastasis of carcinomas, as well as in driving their acquisition of chemoresistant phenotypes [46, 47]. Clinically, elevated expression of miR-21 is a negative predictor for disease-free survival in patients with cancers of the breast or lung [48, 49]. Collectively, these findings illustrate the profound ability of a single miRNA to target multiple oncogenic signaling nodes, resulting in global dysregulation of gene expression networks in carcinoma cells.

#### miR-155

miR-155 was first identified as an oncomiR in B cell lymphomas and chronic lymphocytic leukemias where it functions to accelerate Myc-mediated lymphomagenesis [50, 51]. More recently, miR-155 also appears to be prominently upregulated in solid tumors, including those of the breast, colon, lung, pancreas, and thyroid (*see* [52]). miR-155 transcription is induced by TGF-β, interferon-γ (IFNγ), and interleukin-6 (IL-6) and influences numerous cancer cell signaling pathways to promote tumorigenesis. For instance, by targeting the Rho GTPase, RhoA, miR-155 drives the dissolution of tight junctions that occur during EMT programs and breast cancer invasion stimulated by TGF-β [53]. Likewise, miR-155 also targets and downregulates the expression of the tumor suppressor SOCS1 (suppressor of cytokine signaling 1), leading to constitutive activation of the proto-oncogene STAT3 and its ability to induce inflammatory and proliferative phenotypes in mammary tumors [54]. Finally, miR-155 negates apoptotic signals and enhances cancer cell survival by repressing the expression of FOXO3a, thereby suppressing the expression of the pro-apoptotic proteins PUMA, Bim, FasL, and TRAIL [55].

### Tumor suppressive miRNAs

#### miR-15a and miR-16-1 cluster

The miR-15a and miR-16-1 cluster of miRNAs were initially identified as the most frequently downregulated collection of miRNAs in chronic lymphocytic leukemias [56], an event that also occurs in a subset of multiple myelomas, mantle cell lymphomas, and prostate cancers [57]. Indeed, engineering human MEG-01 chronic myelogenous leukemia cells to overexpress miR-15a~16-1 dramatically suppressed their tumor forming ability when xenografted into nude mice [58]. Interestingly, genome-wide transcriptome profiling analyses indicate the miR-15a~16-1 cluster directly or indirectly regulates as much as 14% of the human genome, particularly for mRNAs housing AU-rich elements (AREs) [58]. These analyses also identified a signature of 60 genes whose expression is downregulated by the miR-15a~16-1 cluster, which preferentially targets genes operant in activating cell cycle progression and survival pathways [58]. Along these lines, intratumoral delivery of miR-15a and miR-16-1 to prostate tumor xenografts induced their regression *via* apoptosis programs that commenced following miRNA-directed downregulation of Bcl-2, cyclin D1, and WNT3A [59]. Collectively, these findings highlight the potent tumor suppressing activities mediated by restoration of the miR-15a~16-1 cluster.

#### Let-7 family

The let-7 family of miRNAs was originally discovered as negative regulators of cell cycle progression and inducers of terminal differentiation in *C. elegans*, and is comprised of 11 homologous miRNAs, including let-7a-1, let-7a-2, let-7a-3, let-7b, let-7c, let-7d, let-7e, let-7f-1, let-7f-2, let-7g, and let-7i [60]. Initial evidence linking the expression of let-7 family members to the process of tumor suppression was obtained in studies of lung carcinomas, which were determined to house significantly reduced expression of let-7 as compared to adjacent normal tumor tissues, an event correlated with diminished overall survival of lung cancer patients [61]. Subsequent studies have clearly established reduced let-7 expression to coincide with the development of cancers of the colon, breast, and ovary [62]. Indeed, overexpression of let-7 miRNAs in breast cancer tumor-initiating cells (TICs) dramatically reduced their ability to proliferate and form mammospheres, as well as enhanced their differentiation status. More importantly, these same let-7 manipulations significantly inhibited the growth and metastasis of breast cancer TICs in mice, doing so *via* let-7-mediated targeting of H-Ras, which negatively impacted TIC self-renewal, and HMGA2, which positively impacted TIC differentiation status [63]. Likewise, restoring let-7 expression in lung carcinoma cells reinstated their sensitivity to radiotherapy *in vitro,* while similarly enhanced or reduced expression of let-7 in *C. elegans* elicited sensitivity or resistance, respectively to radiation-induced cell death [64]. Functionally, oncogenic activation of c-Myc represses let-7 expression [65], while the converse scenario involving the elevated expression and activity of let-7 in human malignancies elicits downregulation of Ras family members [66], HMGA2 [67], and c-Myc [65], thereby suppressing the development, progression, and therapeutic resistance of human tumors.

#### miR-29 family

The miR-29 family contains three members, miR-29a, miR-29b, and miR-29c, all of which are transcriptionally induced by p53 [68] and repressed by c-Myc, NF-κB, and TGF-β signaling (*see* [69]). Functionally, miR-29 serves to reduce cell proliferation by targeting CDK6 [70, 71], to induce cell senescence or differentiation by targeting Ppmid [68] or osteonectin [72], respectively, and to stimulate cell apoptosis by targeting Mcl1 and Bcl2 [73, 74]. Additionally, miR-29 family members have also been observed to target the methyltransferase, DNMT3, leading to hypomethylated promoter regions of a variety of tumor suppressor genes in cancer of the lung, skin, and myeloid compartment [75]. Finally, expression levels of the miR-29 family are inversely correlated with the EMT status of carcinomas, presumably due to the ability of this miRNA family to target extracellular matrix (ECM) proteins operant in driving carcinoma cell migration and metastasis [76]. Thus, in addition to its role in countering malignant transformation, future studies also need to elucidate the extent to which expression of the miR-29 family also functions to suppress the metastatic progression of late-stage carcinomas.

#### miR-34 family

miR-34a and miR-34b/c represent the most highly upregulated miRNAs induced by the tumor suppressor, p53 [77, 78]. Once expressed, these miRNAs function to suppress the growth and metastasis of tumors by promoting apoptosis, cell cycle arrest, and senescence, doing so by targeting and downregulating the expression of oncogenic effectors, including Bcl2, cyclins D1 and E2, CDKs 4 and 6, c-Myc, MET, N-Myc, and SIRT1 [78–83]. Additionally, miR-34 expression is inactivated through epigenetic methylation of its promoter, a reaction that dominates over its transactivation by p53 and occurs in a variety of human malignancies, including those arising in the skin (63%), bladder (33%), lung (29%), breast (25%), kidney (21%), pancreas (16%), and colon (13%) [84]. Likewise, loss of miR-34 expression in prostate cancers has been linked to their acquisition of chemoresistant phenotypes [80]. Collectively, these findings suggest that developing and implementing novel measures capable of re-expressing miR-34 in human carcinomas may provide a unique approach to alleviate metastatic progression and disease recurrence.

### MetastamiRs

The acquisition of metastatic phenotypes by developing carcinomas represents the greatest barrier to effective treatment and long-term survival of cancer patients, of which ~90% succumb to the presence of incurable primary and recurrent metastases [85, 86]. The intractability of metastases likely reflects the complex cascade and series of events necessary for carcinoma cells to disseminate and thrive beyond the confines of the primary tumor. Indeed, navigating the metastatic cascade requires carcinoma cells to *(i)* invade into and migrate through the supporting tumor stroma; *(ii)* intravasate into and traverse throughout the lymph and circulatory systems; and *(iii)* extravasate out of the circulation and ultimately infiltrate and colonize the secondary organ site [85–87]. Although a thorough understanding of the cellular and molecular mechanisms responsible for metastasis remain to be elucidated, recent findings have nevertheless identified a subclass of miRNAs whose expression is highly associated with the acquisition of metastatic phenotypes. Indeed, the importance miRNAs to drive metastasis is highlighted by the fact that diminished Dicer function elicited by miR-103/107 targeting was found to significantly enhance the metastatic activity of mammary tumors [88]. In the succeeding sections, we will discuss a variety of metastasis-related miRNAs, which are referred to as “metastamiRs” and endowed with either metastasis promoting or suppressing activities [89, 90].

#### miR-200 family

The miR-200 family is comprised of 5 related miRNAs, namely miR-200a, miR-200b, miR-200c, miR-141, and miR-429. Functionally, this family serves to maintain epithelial cell gene expression profiles, morphologies, and characteristics, thereby suppressing the acquisition of EMT and metastatic phenotypes [91, 92]. Mechanistically, miR-200 family members promote E-cadherin expression by repressing that of the master EMT transcription factors, Zeb1 and Zeb2, which function in preventing the production of E-cadherin transcripts [93]. Accordingly, enforced expression of miR-200 is sufficient to alleviate the ability of lung cancers to undergo EMT programs and, consequently, to engage in invasion and metastatic behaviors in mice [94]. Interestingly, recent studies have associated metastasis-promoting activities with the expression of miR-200 family members in the late-stages of metastatic colonization and outgrowth, doing so by downregulating the expression of Sec23a and preventing its secretion of the metastasis suppressing proteins, IGFBP4 (insulin-like growth factor-binding protein 4) and TINAGL1 (Axl receptor tyrosine kinase, tubulointerstitial nephritis antigen-like 1) [95]. Collectively, these findings indicate that the ability of miR-200 family members to either suppress (*e.g.,* early-stage disease) or promote (*e.g.,* late-stage disease) metastasis transpires in a context-dependent manner that may reflect alterations in the genetic landscape of developing carcinomas as they progress from early-stages to late-stages of the disease.

#### miR-9

In stark contrast to members of the miR-200 family and their indirect coupling to E-cadherin expression *via* Zeb1/2, the expression of miR-9 serves to promote invasive and metastatic phenotypes by directly downregulating the levels of E-cadherin transcripts in mammary carcinomas [96]. In addition to its ability to suppress the expression of E-cadherin, miR-9 also promotes carcinoma invasion and metastasis by targeting the leukemia inhibitory factor receptor (LIFR), leading to the inactivation of prometastatic signals mediated by the Hippo-YAP pathway [97]. Interestingly and reminiscent of the context-specific activities of miR-200 family members, Zheng *et al.* [98] recently observed the expression of miR-9 to be dramatically downregulated in gastric cancers, an event associated with their acquisition of proliferative, invasive, and metastatic phenotypes. Importantly, the tumorigenicity of gastric cancers was significantly suppressed by restoring miR-9 expression in miR-9-deficient gastric cancers, thereby reducing the expression of the cyclin D1 and Ets1 oncogenes [98]. Thus, future studies need to determine the contexts and genetic backgrounds that underlie the gain or loss of miR-9 expression in human malignancies, as well as how these events contribute to the growth and metastatic progression of the neoplastic lesions.

#### miR-31

The expression of miR-31 is inversely correlated with the metastatic capacity of more than 15 breast cancer cell lines [99], leading to its designation as a metastasis-suppressing miRNA. Accordingly, re-expression of miR-31 in human breast cancers that lack expression of this miRNA significantly impaired their metastatic dissemination in mice [99], doing so by inhibiting breast cancer migration and invasion, and by sensitizing these same cells to anoikis-mediated apoptosis [99]. Mechanistically, miR-31 suppresses metastasis by targeting the expression of α5 integrin (ITGA5), radixin (RDX), RhoA, and WAVE3 (WAS protein family member 3) [100, 101]. Clinically, abnormally low miR-31 expression levels in primary breast tumors is associated with increased metastasis and disease relapse [99, 101], indicating that miR-31 does indeed act as a potent inhibitor of the metastatic cascade.

#### miR-10b

The finding that miR-10b is differentially expressed and significantly higher in metastatic breast cancer cells as compared to their nonmetastatic counterparts suggest that this miRNA promotes the acquisition of metastatic phenotypes in developing carcinomas [102, 103]. Indeed, tumor specimens obtained from breast cancer patients demonstrated that elevated miR-10b expression was prevalent in patients harboring metastatic disease [102]. Moreover, abnormally elevated expression of miR-10b correlates with the appearance of high-grade malignancies in the liver, pancreas, and brain [104]. Consistent with its designation as a “metastamiR”, overexpression of miR-10b preferentially drives the metastasis of breast and esophageal cancers in mice without effecting the behaviors of the corresponding primary tumors [102, 105]. Mechanistically, miR-10b expression is upregulated by the EMT-promoting transcription factor, Twist, which results in miR-10b-mediated suppression of HOXD10 expression and, consequently, in the upregulated expression of the cell motility and ECM remodeling genes RhoC, uPAR (urokinase-type plasminogen activator receptor), α3 integrin, and MT1-MMP (membrane-type 1 MMP) [102]. Finally, systemic delivery of miR-10b antagomiRs potently inhibited the metastasis of breast cancers in mice, an event that occurred with little-to-no measurable toxicity [106], suggesting that inactivation of miR-10b expression and activity holds potential to prevent carcinoma metastasis.

#### miR-181a

miR-181a has been shown to function as either a tumor suppressor or a tumor promoter in a context-dependent manner (*see* [107]). For instance, we recently uncovered a novel role for miR-181a in promoting breast cancer metastasis and showed that the expression of this metastamiR correlates with the metastatic potential of breast cancers, particularly those classified as triple-negative (*i.e.,* lack ER-α and progesterone receptor expression, and fail to amplify HER2) [108]. Functionally, upregulated miR-181a expression was observed to inhibit anoikis by targeting the pro-apoptotic protein, Bim, and to enhance EMT programs stimulated by TGF-β [108]. Likewise, miR-181a expression has been linked to the generation and expansion of cancer stem cells through its ability to target ATM [109]. Importantly, targeted inactivation of miR-181a reduced the metastatic outgrowth of breast cancer cells in mice [108], a finding consistent with the demonstration that high miR-181a levels correlated with a dramatic decline in the overall survival of breast cancer patients [108]. Along these lines, upregulated miR-181a expression in epithelial ovarian cancers enhanced TGF-β signaling and disease progression by targeting the inhibitory Smad, Smad7, leading to the acquisition EMT, motile, and chemoresistant phenotypes in caners of the ovary [110]. In stark contrast, loss of miR-181a expression suggestive of a tumor suppressive function has been detected in a variety of human cancer cell lines, including those derived from the lung and brain [107]. Although the mechanisms responsible for eliciting discrepant miR-181a expression profiles remains to be fully elucidated, recent findings implicate expression of p53 as a molecular determinant of miR-181a expression [107]. As such, future studies need to better understand the relationship between miR-181a expression and that of classical tumor suppressors and promoters before undertaking the therapeutic targeting of miR-181a to alleviate carcinoma metastasis.

### Therapeutic strategies for targeting miRNAs

The small size, extreme stability, and potent biological activities exhibited by miRNA oligonucleotides suggests that measures capable of targeting and/or delivering miRNAs to developing carcinomas holds great promise to alleviate disease progression and improve overall survival rates of cancer patients. Accordingly, the pharmacologic delivery of miRNA oligonucleotides or viral-based miRNA expression constructs to tumors seeks limit their growth and dissemination *via* 3 general strategies: *(i)* inactivate the oncogenic activities of oncomiRs; *(ii)* reinstate the expression of tumor suppressive miRNAs; and *(iii)* administer chemotherapeutic agents capable of regulating miRNA transcription or processing.

### Anti-oncomiR strategies

#### Antisense oligonucleotides

Antisense oligonucleotides inhibit target miRNAs by annealing to complementary sequences within mature miRNAs, thereby inducing their degradation or blocking their function. The inherent instability of single-stranded oligonucleotides has been circumvented by the incorporation of chemical modifications designed to increase the stability, binding affinity, and specificity of newly synthesized single-stranded oligonucleotides [111]. For instance, adding 2′-*O*-methyl modifications increases the stability of nucleotides [112], while adding sulphur atoms to form phosphorothiote bonds further increases nucleotide stability, but often at the expense decreasing binding affinity [113]. More recently, 2′-*O*-methyoxyethyl (2′-MOE) and 2′-Fluoro (2′-F) modifications have been shown to provide superior binding affinity as compared to 2′-*O*-methyl modifications [114, 115]. Thus, selective modification of both the 2′ sugar position and phosphodiester bonds is necessary to obtain optimal nucleotide stability and affinity. Indeed, single-stranded phosphorothioate-linked RNA analogues that contain 2′-O-methyl-modified cholesterol-conjugated nucleotides have been shown to be effective in specifically targeting miR-122 in the liver for 23 days following a single intravenous injection of the ‘miR-122 antagomiR’ [113].

#### Locked nucleic acid (LNA) constructs

Locked nucleic acid (LNA) constructs represent a novel class of nucleic acid analogs characterized by the presence of a “locked” ribose ring generated by the formation of a methylene bridge between the 2′-O atom and the 4′-C atom [116]. Interestingly, antisense compounds that house a “locked” configuration display several desirable features as compared to traditional “antagomiR” chemistry, including *(i)* increased binding affinity against complementary single stranded RNA molecules, *(ii)* enhanced discrimination against base mismatches, and *(iii)* improved efficiency of target miRNA knockdown and inactivation [116]. More recently, LNA chemistry served as the foundation during the synthesis of the anti-miR-122 agent developed by Santaris Pharma (SPC3649), which is highly effective in downregulating miR-122 levels in the liver in non-human primates (African Green Monkeys; [117]), and in treating chronic HCV infection in chimpanzees [118]. In both scenarios, administration of SPC3649 was demonstrated to possess low toxicity profiles, indicating that LNA molecules are well-tolerated *in vivo* and, consequently, may represent ideal agents designed to alleviate the tumor promoting activities of oncomiRs in cancer patients.

#### miRNA sponges and masks

miRNA sponges serve as competitive inhibitors by binding complementary miRNAs, thereby ‘soaking up’ available oncomiR reservoirs. Typically, miRNA sponges are retroviral vectors engineered to house multiple tandem miRNA binding sites whose expression is driven from a strong promoter, resulting in the supraphysiological expression of desired “antagomiRs”. At present, stable expression of miRNA sponges, particularly those that house fluorescent reporter genes within the vector backbone have proven to be valuable research tools to study the impact of miRNA loss-of-function scenarios in animal models of carcinoma development and metastatic progression. Unfortunately, the necessity of miRNA sponges to be stably expressed within target cells has greatly limited their overall therapeutic value due to issues related to the delivery of miRNA sponge retroviral particles specifically and efficiently to carcinoma cells, not normal host [119].

In stark contrast to the mechanism of action of miRNA sponges, miRNA masks antagonize miRNA function by binding directly to 3'-UTR sequences in target mRNAs, thereby protecting their integrity by preventing miRNA binding reactions [120]. Experimentally, this technique provides an innovative approach to determine the extent to which a given cellular phenotype is driven by distinct miRNA:mRNA pairs. Clinically, however, this technique is also limited by drug delivery and specificity issues, as well as by the fact that targeting and/or protecting a single mRNA *via* miRNA masks is unlikely to outperform the delivery of miRNA sponges, which will restore and/or protect the expression of hundreds of mRNAs in carcinoma cells.

### miRNA overexpression strategies

“miRNA replacement therapy” has been proposed as a novel means to restoring tumor suppressing miRNAs at supraphysiological levels with developing and progressing carcinomas, doing so by engineering and delivering double-stranded 2′O-methyl phosphorothioate-containing miRNA mimics. Indeed, the growth of lung tumors in mice was severely compromised by the delivery of miRNA mimics for let-7b and miR-34 [121], as was that of prostate tumors following the administration of miRNA mimics for miR-15a and miR-16 [59]. Alternatively, the use of adenovirus-associated vectors (AVV), which do not integrate into the genome and are eliminated with minimal toxicity [122, 123], have also been employed to deliver and express tumor suppressing miRNAs in carcinomas. Indeed, systemic administration of AAV-miR-26a viral particles to mice bearing hepatocellular carcinomas resulted in a dramatic reduction in disease progression due to decreased cell proliferation and survival [124]. However, due to the extensive alterations that transpire within the miRNA processing system in malignant cells, it may prove to be especially challenging to achieve sustained therapeutic expression levels of tumor suppressing miRNAs in carcinomas, particularly over long periods of time and treatment durations.

### Small-molecule inhibitors

Numerous small molecule inhibitors have been shown to elicit global alterations in miRNA expression profiles [5]. For instance, the administration of small molecule inhibitors against specific oncogenic effectors, transcription factors, or pathways can suppress the upregulated expression of oncomiRs. Alternatively, it may be feasible to develop novel small molecules capable of altering miRNA biogenesis, thereby impacting global miRNA expression profiles. Indeed, Gumireddy *et al.* [125] recently screened a luciferase-miR-21 reporter gene against chemical library that contained more than 1000 compounds, resulting in the identification of 2 small molecule inhibitors against miR-21 and that prevent its binding to 3′UTR seed sequences. Expanding similar miRNA-based reporter screens to larger and more complex chemical libraries, as well as to those comprised of FDA-approved drugs (*i.e.,* drug repurposing) holds tremendous potential in isolating a variety of novel agents capable of inhibiting miRNA function both experimentally and clinically.

### miRNAs as biomarkers

miRNAs are normally expressed in a developmental- and tissue-specific manner to maintain cell and tissue homeostasis [126]. However, disease development disrupts this cellular equilibrium and elicits aberrant miRNA expression in a disease-specific manner, particularly with respect to genetically distinct subtypes of breast cancer. In fact, miRNA signatures have recently been observed to possess more predictive power as compared to their larger and more extensive mRNA signature counterparts [127]. Along these lines, miRNA profiles are more effective in identifying tumors of unknown origin than their corresponding mRNA profiles [126]. Likewise, monitoring the presence of circulating miRNAs has recently been undertaken as a means to readily distinguish cancer patients from healthy individuals, and to predict overall and relapse-free survival rates with extreme accuracy [128]. Given the remarkable stability of miRNAs in the circulation, tissues, and other biological fluids, future studies clearly need to expand our understanding and repertoire of miRNA signatures and their specificity for diagnosing human malignancies, and for monitoring carcinoma development, metastatic progression, and recurrence.

## Conclusions

The ability of miRNAs to function as tumor promoters or tumor suppressors is well-established, as is their role in regulating normal tissue homeostasis and disease development in humans. Moreover, the recent success of miRNA-based therapies to treat HCV, coupled with the apparent effectiveness of miRNA-targeted oligonucleotides to alleviate tumor development in preclinical models of cancer, suggests that miRNAs represent a feasible class of targets to treat human malignancies. Likewise, pharmacological targeting of miRNAs will impact a plethora of oncogenic signaling nodes and biological processes due to the extreme range of mRNAs governed by a single miRNA, thereby offering a unique therapeutic advantage over current single gene/molecule-based approaches to cancer. However, the broad spectrum of genes targeted by miRNA-based therapeutics might also present a number of challenges, particularly the potential for off-target miRNA activities that could lead to unwanted toxicities. As such, improving miRNA target prediction algorithms and garnering a greater understanding of miRNA signaling networks will aid significantly in the development of highly specific and safe miRNA-targeted therapies. Equally challenging are overcoming the difficulties associated with the administration and effective delivery miRNA cargos to tumor tissues. Although recent chemical modifications to RNA-based structures have greatly improved their stability and lessened their toxicities, the ability to target and deliver similarly modified miRNAs to tumors may nevertheless prove to be daunting undertaking. Thus, while the packaging of miRNAs within nanoparticles and liposomes may indeed improve their ultimate delivery to developing tumor microenvironments, it is clear that future studies need to elucidate the optimal chemistry profiles necessary to maximize oligonucleotide delivery to malignant tissues and cells, thereby alleviating the development, metastasis, and recurrence of human carcinomas.

## Abbreviations

Ago: Argonaute
BMP: Bone morphogenetic protein
CDK: Cyclin-dependent protein kinase
DNMT3: DNA (cystosine-5-)-methyltransferase 3
HCV: Human cytomegalovirus
EMT: Epithelial-mesenchymal transition
HOXD10: Homeobox D10
IFNγ: Interferon-γ
IL-6: Interleukin-6
ITGA3: α3 integrin
ITGA5: α5 integrin
HMGA2: High mobility group AT-hook 2
LIFR: Leukemia inhibitory factor receptor
lncRNA: Long noncoding RNA
miRNA: MicroRNA
MMP: Matrix metalloproteinase
MT1-MMP: Membrane-type 1 MMP
NF-κB: Nuclear factor-κB
RDX: Radixin
SBE: Smad-binding element
TGF-β: Transforming growth factor-β
TIC: Tumor-initiating cell
Tsp1: Thrombospondin-1
uPAR: Urokinase-type plasminogen activator receptor
3′UTR: 3′-untranslated region
WAVE3: WAS protein family member 3.
